# MRI-derived extracellular volume as a biomarker of cancer therapy cardiotoxicity: systematic review and meta-analysis

**DOI:** 10.1007/s00330-023-10260-8

**Published:** 2023-10-12

**Authors:** Gianluca Folco, Caterina B. Monti, Moreno Zanardo, Francesco Silletta, Davide Capra, Francesco Secchi, Francesco Sardanelli

**Affiliations:** 1 Postgraduation School in Radiodiagnostics, University of Milan, Milan, Italy; 2 Department of Biomedical Sciences for Health, University of Milan, Milan, Italy; 3 Unit of Radiology, IRCCS Policlinico San Donato, San Donato Milanese, Italy

**Keywords:** Myocardium, Cardiotoxicity, Magnetic resonance imaging, Meta-analysis

## Abstract

**Objectives:**

MRI-derived extracellular volume (ECV) allows characterization of myocardial changes before the onset of overt pathology, which may be caused by cancer therapy cardiotoxicity. Our purpose was to review studies exploring the role of MRI-derived ECV as an early cardiotoxicity biomarker to guide timely intervention.

**Materials and methods:**

In April 2022, we performed a systematic search on EMBASE and PubMed for articles on MRI-derived ECV as a biomarker of cancer therapy cardiotoxicity. Two blinded researchers screened the retrieved articles, including those reporting ECV values at least 3 months from cardiotoxic treatment. Data extraction was performed for each article, including clinical and technical data, and ECV values. Pooled ECV was calculated using the random effects model and compared among different treatment regimens and among those who did or did not experience overt cardiac dysfunction. Meta-regression analyses were conducted to appraise which clinical or technical variables yielded a significant impact on ECV.

**Results:**

Overall, 19 studies were included. Study populations ranged from 9 to 236 patients, for a total of 1123 individuals, with an average age ranging from 12.5 to 74 years. Most studies included patients with breast or esophageal cancer, treated with anthracyclines and chest radiotherapy. Pooled ECV was 28.44% (95% confidence interval, CI, 26.85−30.03%) among subjects who had undergone cardiotoxic cancer therapy, versus 25.23% (95%CI 23.31−27.14%) among those who had not (*p* = .003).

**Conclusion:**

A higher ECV in patients who underwent cardiotoxic treatment could imply subclinical changes in the myocardium, present even before overt cardiac pathology is detectable.

**Clinical relevance statement:**

The ability to detect subclinical changes in the myocardium displayed by ECV suggests its use as an early biomarker of cancer therapy–related cardiotoxicity.

**Key Points:**

*• Cardiotoxicity is a common adverse effect of cancer therapy; therefore, its prompt detection could improve patient outcomes.*

*• Pooled MRI-derived myocardial extracellular volume was higher in patients who underwent cardiotoxic cancer therapy than in those who did not (28.44% versus 25.23%, p = .003).*

*• MRI-derived myocardial extracellular volume represents a potential early biomarker of cancer therapy cardiotoxicity.*

**Supplementary Information:**

The online version contains supplementary material available at 10.1007/s00330-023-10260-8.

## Introduction

Mortality from most types of cancer has decreased considerably in recent years, as a result of the improvements in screening programs and treatment efficacy [1]. However, cancer therapy still carries a significant burden of side effects, among which cardiovascular complications arising from non-reversible cardiotoxicity present a major concern due to their high morbidity and mortality [2]. The main treatments associated with cardiotoxicity are conventional chemotherapeutic agents such as anthracyclines, chest radiotherapy, and targeted therapies such as monoclonal antibodies and small molecule inhibitors [3].

Cancer therapy-related cardiac dysfunction is defined as a decline of at least 10% in left ventricular ejection fraction (LVEF) [4]. The 2022 European Society of Cardiology (ESC) guidelines on Cardiooncology recommend assessment of LVEF and myocardial strain at echocardiography for the detection of cancer therapy–related toxicity, along with monitoring of relevant serum biomarkers [5]. However, as the heart presents a significant functional reserve, substantial damage to cardiomyocytes may occur before an overt reduction in LVEF [6]. Over the years, several potential biomarkers have been proposed, but none so far has yielded high accuracy for detection of subtle myocardial changes before overt heart failure in clinical practice [7].

In recent years, parametric mapping techniques from cardiac MRI have emerged as tools to assess myocardial tissue composition [8]. In particular, T1 mapping techniques can provide T1 relaxation times for the myocardium before and after the intravenous administration of extracellular gadolinium-based contrast agents, allowing to estimate cardiac extracellular volume (ECV) on a voxel-by-voxel basis [9]. Increases in T1 relaxation times are expected in case of myocardial edema or fibrosis [10], which are the macroscopic signs of cellular death following apoptosis and necrosis. Similarly, as the ECV reflects the percentage of the heart that is not composed by cells, it is also expected to increase in the presence of edema or extracellular protein deposition also in absence of cellular death [11].

The T1 mapping–derived estimation of ECV may thus represent an emerging biomarker that allows characterization of myocardial composition, its value rising in conditions of myocardial inflammation or fibrosis [12], in good correlation with histopathological findings [13]. As cardiotoxicity from cancer therapy is represented by cardiomyocyte death that ultimately leads to tissue fibrosis, ECV may warrant an early, accurate detection of subtle changes in the myocardial tissue, allowing physicians to undertake preventive measures to avoid overt cardiotoxicity. For instance, detecting subclinical cardiotoxicity in patients undergoing anthracycline-based chemotherapy regimens may lead to the initiation of therapeutical adjustments while continuing anthracycline chemotherapy, such as pre-treatment with dexrazoxane before each therapy cycle, and personalized follow-up schemes.

Therefore, the purpose of this systematic review and meta-analysis was to investigate the studies exploring the role of ECV as a biomarker of cardiotoxicity from cancer therapy, to better understand its potential in this clinical setting.

## Materials and methods

### Search strategy and eligibility criteria

Ethics committee approval was not required for this systematic review and meta-analysis. We registered our systematic review and meta-analysis on ResearchGate (https://www.researchgate.net/project/Extracellular-volume-fraction-as-an-MRI-biomarker-of-chemotherapy-cardiotoxicity-a-systematic-review), and it was reported according to the Preferred Reporting Items for Systematic reviews and Meta-Analyses (PRISMA) statement [14].

In April 2022, we performed a systematic search on EMBASE (Excerpta Medica dataBASE, embase.com) and PubMed (US National Library of Medicine, pubmed.ncbi.nlm.nih.gov) for articles reporting the use of MRI-derived ECV as a cancer therapy–related cardiotoxicity biomarker.

The adopted search string included MeSH terms, and was built using the following strategy, based on the PICO model:

- Problem: ‘extracellular space’/exp + synonyms
- Intervention: ‘cardiovascular magnetic resonance’/exp + synonyms
- Comparison condition (exposure, risk/prognostic factor) ‘chemotherapy’/exp OR ‘radiotherapy’/exp + synonyms
- Outcome: ‘cardiotoxicity’/exp + synonyms

Full search strings are reported in Supplementary Material 1. The search was limited to original studies written in English with an available abstract, performed on human subjects, and published either on paper or online on peer-reviewed journals. No limits were applied to publication date. Identical duplicate records which had already been retrieved from EMBASE were not included among those retrieved via PubMed.

### Data extraction

Two blinded researchers (G.F. and F.Si.), both with 2 years of experience in cardiovascular imaging, performed an initial screening of the retrieved articles, based on title and abstract only. All selected articles, including those with abstracts lacking complete information to determine inclusion/exclusion criteria, were then downloaded and, after a blinded full-text screening by each researcher, only those reporting MRI-derived ECV values at least 3 months after cardiotoxic cancer therapy were included. Disagreements were discussed by the two researchers in consensus and, whenever no agreement was reached, a third reader (C.B.M.) acted as arbiter. Lastly, references from the included articles that could potentially meet the inclusion criteria were subsequently manually screened.

The same researchers who performed the literature search independently extracted all data using a standardized datasheet, and disagreements were resolved by consensus. Studies with overlapping patient cohorts were excluded. For each included article, when available, the following data were extracted: year of publication and country of origin, study design (prospective or retrospective), population demographics and clinical data (e.g., gender and LVEF), type of malignancy, treatment regimen, MRI acquisition time from treatment, MRI protocol, and ECV values. Study parts were labeled as referring to cases or controls when patients had or had not undergone cardiotoxic cancer therapy regimens, respectively. Study parts including patients with previous cardiac comorbidities (e.g., hypertrophic cardiomyopathy) were not considered, to avoid a confounding effect on ECV values; moreover, we excluded study parts for which complete treatment regimen was not clearly specified, as their cardiotoxic potential could not be correctly assessed.

### Quality assessment

Two researchers (M.Z. and C.B.M.), with 5 and 4 years of experience in cardiovascular imaging, assessed the quality of the included articles in consensus, using the Standard Quality Assessment Criteria (QualSyst tool) [15].

### Statistical analysis

Statistical analysis was performed using R (version 4.2.1, R Foundation for Statistical Computing) on RStudio (version 1.1.456, RStudio PBC). The R package “readxl” [16] was used to import extracted data, whereas the package “meta” [17] was used to perform the meta-analysis. Due to significant heterogeneity of ECV values reported by different studies, pooled ECV was calculated using the random effects model, the DerSimonian-Laird estimator [18], with the Knapp-Hartung-Sidik-Jonkman adjustment [19], in subjects who had or had not undergone cardiotoxic cancer therapy, respectively. Pooled ECV was also compared among different treatment regimens, and among those who did or did not experience overt cardiac dysfunction, via post hoc analyses. Meta-regression analyses were conducted to appraise which clinical or technical variables yielded a significant impact on ECV, and differences among those who had or had not undergone cardiotoxic cancer therapy were appraised for those variables that did, via post hoc analyses. Moreover, for those studies including both a case and control group, standardized mean differences were calculated and meta-analyzed as previously described. The risk of publication bias was evaluated via both funnel plots and the Egger test [20]. The threshold for statistical significance was set at *p* ≤ .05 [21].

## Results

### Study selection

**Fig. 1:**
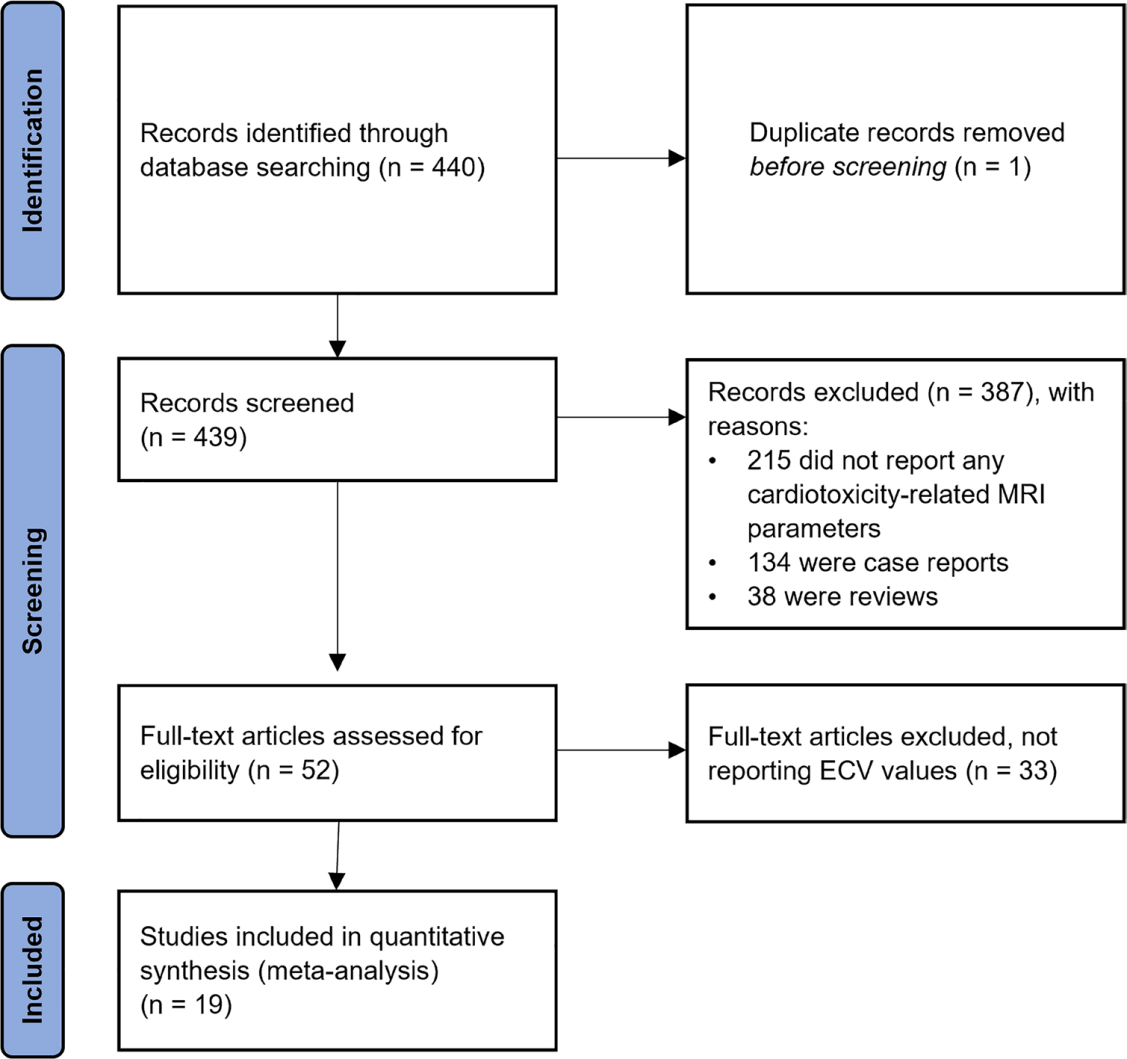
Flowchart outlining the study selection process

The flowchart depicting study selection is shown in Fig. 1. From 439 initially retrieved individual articles, 52 were included after the first selection based on article title and abstract. Out of all the excluded articles, 215 did not include MRI-derived parameters after cardiotoxic treatment, 134 were case reports, and 38 were reviews. Out of the 52 articles included at the first selection, 33 did not report post-treatment ECV values in the full text, leading to a final number of 19 included papers. A total of 29 study parts, including both cancer survivors and healthy controls, were eligible for meta-analysis.

### Data extraction

Included works (22–40) were published between 2013 [22, 23] and 2022 [24, 25], and all but 2 [26, 27] had a prospective design. Six studies were conducted in the USA [22, 25, 28–31], 4 in Canada [23, 32–34], 4 in Germany [27, 35–37], 2 in the Netherlands [24, 38], 1 in the UK [26], 1 in Norway [39], and 1 in Japan [40].

Study population for each study part ranged from 9 [37] to 236 [31] patients, for a total of 1123 enrolled individuals. The average age of patients in each study part ranged from 12.5 [29] to 74 [24] years.

Six studies included only patients with breast cancer [28, 30, 32, 34, 35, 39], 3 studied patients with esophageal cancer [24, 38, 40], and 1 included patients with sarcoma [37], while the others included patients with mixed types of neoplasms, most frequently breast, lung, and hematological malignancies.

Concerning cancer therapy, 13 study parts analyzed the cardiotoxic effects of anthracyclines [22, 23, 26, 27, 29–31, 33, 37, 39], and 3 study parts focused on the combination of anthracyclines and antibodies [32, 34], 3 on the combination of chest radiotherapy and anthracyclines [25, 28, 35], and 3 on chest radiotherapy coupled to non-cardiotoxic regimens [24, 38, 40], while 1 study part focused solely on the effects of chest radiotherapy [35] and 1 on the effects of antibodies [36].

Scans were performed on 1.5-T (22 study parts) and 3-T (7 study parts) systems. Clinical and technical data for each study part, including time from treatment and MRI protocol, are reported in Tables 1 and 2, respectively.

**Table 1:** Clinical data from the included works. Different study parts are labeled with letters

Study name	Cardiotoxic treatment	Country	Cancer	Treatment regimen	Design	*N*	*F*	Age (years)	Months from treatment	CTRCD	ECV (%)	ECV post (%)	MRI–LVEF (%)	MRI–LVEF post (%)
Beukema et al 2022	Y	The Netherlands	Esophagus	RT + non-cardiotoxic chemotherapy	P	20		67.8	88*	N		28.4 ± 0.3		
Beukema et al 2022	N	The Netherlands	Esophagus	Surgery only	P	20		74	126*	N		24 ± 0.3		
Canada et al 2022	Y	USA	Lung, breast, other chest malignancies	RT + anthracyclines	P	27		63	24*	N		28		64
de Groot et al 2021	Y	The Netherlands	Esophagus	RT + non-cardiotoxic chemotherapy	P	17	6	67.6 ± 8.1	87 ± 23*	N		28.4 ± 1		57.9 ± 13.6
de Groot et al 2021	N	The Netherlands	Esophagus	Surgery only	P	16	3	71.8 ± 9.6	122 ± 35*	N		24 ± 0.9		57.4 ± 7.8
Tahir et al 2021	Y	Germany	Breast	RT + anthracyclines	P	38	38	51 ± 11	13 ± 2^#^	N	28 ± 2	29 ± 2	60 ± 5	60 ± 6
Tahir et al 2021	Y	Germany	Breast	RT	P	27	27	56 ± 14	13 ± 1^#^	N	30 ± 3	30 ± 3	62 ± 5	62 ± 5
Harries et al 2021	Y	UK	Hematological, breast	Anthracyclines	R	45	27	56 ± 16	11*	N		29.5 ± 4.5		59.5 ± 4.1
Harries et al 2021	N	UK	None	None	R	45	27	53 ± 16		N	27.4 ± 2.3	27.4 ± 2.3	60.8 ± 2.4	60.8 ± 2.4
Kirkham et al 2021	Y	Canada	Breast	Anthracyclines + antibodies	P	94		51 ± 8	12^#^	N	22.9 ± 3.3	22.4 ± 3.5		
Faron et al 2021	Y	Germany	Melanoma, squamous cell carcinoma, lung	Antibodies	P	22	9	65 ± 14	3.6 ± 1^#^	N	25.6 ± 4.5	26 ± 3.8	62 ± 7	59 ± 7
Mawad et al 2021b	Y	Canada	Pediatric cancer	Anthracyclines	P	48		15.1 ± 2.8	117.6*	N		26.6 ± 7.3		55 ± 5
Mawad et al 2021b	N	Canada	None	None	P	25		14.2 ± 2.4		N	21.7 ± 2.6	21.7 ± 2.6	58 ± 5	58 ± 5
Altaha et al 2020a	Y	Canada	Breast	Anthracyclines + antibodies	P	10	10	54.2 ± 6.6	5.5^#^	N	25.3 ± 1.1	26.1 ± 1.3	62.4 ± 4.1	61.1 ± 3.5
Altaha et al 2020b	Y	Canada	Breast	Anthracyclines + antibodies	P	10	10	52.3 ± 8.5	5.5^#^	Y	23.8 ± 2.3	26.2 ± 3.3	63.9 ± 3.1	51.5 ± 2.3
Altaha et al 2020	N	Canada	None	None	P	30	18	46 ± 13.7		N	24 ± 2.6	24 ± 2.6	61 ± 3.9	61 ± 3.9
Bergom et al 2020	Y	USA	Breast	RT + anthracyclines	P	20	20	59	99.6*	N		27		63
Mokshagundam et al 2020	Y	USA	Pediatric cancer (sarcoma, hematological)	Anthracyclines	P	30	11	12.5	47.7*	N		24.8		58
Wolf et al 2020	Y	Germany	Pediatric cancer (sarcoma, hematological)	Anthracyclines	R	79	36	20.9	134.4 ± 54*	N		22 ± 2		
Ferreira de Souza et al 2018	Y	Brazil	Breast	Anthracyclines	P	27	27	51.8 ± 8.9	17.3^#^	N	32 ± 4	36 ± 4	69.4 ± 3.6	57.5 ± 6.1
Muehlberg et al 2018a	Y	Germany	Sarcoma	Anthracyclines	P	14			5.5^#^	N	26.4 ± 2	29.4 ± 1.6	59.2 ± 10.2	58.3 ± 7.8
Muehlberg et al 2018b	Y	Germany	Sarcoma	Anthracyclines	P	9			5.5^#^	Y	27.5 ± 2.7	29.8 ± 1.7	63.5 ± 5.8	49.9 ± 5
Takagi et al 2018	Y	Japan	Esophagus	RT + non-cardiotoxic chemotherapy	P	21			6.2 ± 0.7^#^	N	27 ± 4	33 ± 3		65 ± 12
Heck et al 2017	Y	Norway	Breast	Anthracyclines	P	69	69			N	27.5 ± 2.7	28.6 ± 2.9	62.8 ± 4.6	61.1 ± 4.4
Jordan et al 2016	Y	USA	Breast, hematological, sarcoma	Anthracyclines	P	37	29	53 ± 13	36 ± 18*	N		30.4 ± 0.7		53 ± 9
Jordan et al 2016	N	USA	None	None	P	236	140	67 ± 9		N	26.9 ± 0.2	26.9 ± 0.2	61 ± 7	61 ± 7
Neilan et al 2013	Y	USA	Hematological, breast, sarcoma	Anthracyclines	P	42	21	55 ± 17	84*	N		36 ± 3		52 ± 12
Neilan et al 2013	N	USA	None	None	P	15	8	56 ± 13		N	28 ± 2	28 ± 2	62 ± 5	62 ± 5
Tham et al 2013	Y	Canada	Pediatric cancer (hematological, sarcoma)	Anthracyclines	P	30	15	15.2 ± 2.7	91.2 ± 54*	N		20.7 ± 3.6		57.6 ± 4.9

*N*°, patients’ number; *F*, females; *CTRCD*, reported group with cancer therapy–related cardiac dysfunction; *ECV*, extracellular volume; *MRI-LVEF*, magnetic resonance imaging–derived left ventricular ejection fraction; *RT*, radiation therapy; *Y*, yes; *N*, no; *P*, prospective; *R*, retrospective. *Months from the end of treatment; ^#^months from the start of treatment

**Table 2:** Technical data from the included works. Different study parts are labeled with letters

Study name	MRI unit	T	Contrast agent	Dose (mmol/kg)	T1 mapping sequence	Timing post contrast (min)
Beukema et al 2022	AvantoFit (Siemens)	1.5	N/A	N/A	N/A	N/A
Beukema et al 2022	AvantoFit (Siemens)	1.5	N/A	N/A	N/A	N/A
Canada et al 2022	Aera (Siemens)	1.5	Gadoteridol	0.2	MOLLI	15
de Groot et al 2021	AvantoFit (Siemens)	1.5	Gadoterate meglumine	0.2	MOLLI	12
de Groot et al 2021	AvantoFit (Siemens)	1.5	Gadoterate meglumine	0.2	MOLLI	12
Tahir et al 2021	Ingenia (Philips)	3	Gadoterate meglumine	0.15	MOLLI	10
Tahir et al 2021	Ingenia (Philips)	3	Gadoterate meglumine	0.15	MOLLI	10
Harries et al 2021	Avanto (Siemens)	1.5	N/A	N/A	MOLLI	N/A
Harries et al 2021	Avanto (Siemens)	1.5	N/A	N/A	MOLLI	N/A
Kirkham et al 2021	Siemens	1.5	Gadopentetate dimeglumine	0.15	SASHA	20
Faron et al 2021	Ingenia (Philips)	1.5	Gadoterate meglumine	0.2	MOLLI	10
Mawad et al 2021b	Avanto (Siemens)	1.5	Gadopentetate dimeglumine	0.2	MOLLI	15
Mawad et al 2021b	Avanto (Siemens)	1.5	Gadopentetate dimeglumine	0.2	MOLLI	15
Altaha et al 2020a	AvantoFit (Siemens)	1.5	Gadobutrol	0.2	MOLLI	15
Altaha et al 2020b	AvantoFit (Siemens)	1.5	Gadobutrol	0.2	MOLLI	15
Altaha et al 2020	AvantoFit (Siemens)	1.5	Gadobutrol	0.2	MOLLI	15
Bergom et al 2020	Verio (Siemens)	3	Gadopentetate dimeglumine	0.2	MOLLI	15
Mokshagundam et al 2020	Aera (Siemens)	1.5	Gadobutrol	0.15	MOLLI + SASHA	15–22
Wolf et al 2020	Avanto (Siemens)	1.5	Gadopentetate dimeglumine	N/A	MOLLI	10
Ferreira de Souza et al 2018	Achieva (Philips)	3	Gadoterate meglumine	0.2	Look–locker	10
Muehlberg et al 2018a	AvantoFit (Siemens)	1.5	Gadoteridol	0.2	MOLLI	15
Muehlberg et al 2018b	AvantoFit (Siemens)	1.5	Gadoteridol	0.2	MOLLI	15
Takagi et al 2018	Tim Trio (Siemens)	3	Gadopentetate dimeglumine	0.15	MOLLI	15
Heck et al 2017	Achieva (Philips)	1.5	Gadoterate meglumine	0.2	MOLLI	15
Jordan et al 2016	Avanto (Siemens)	1.5	Gadopentetate dimeglumine/gadoteridol	0.15/0.2	MOLLI	12
Jordan et al 2016	Avanto (Siemens)	1.5	Gadopentetate dimeglumine/gadoteridol	0.15/0.2	MOLLI	12
Neilan et al 2013	Tim Trio (Siemens)	3	Gadopentetate dimeglumine	0.15	Look–locker	N/A
Neilan et al 2013	Tim Trio (Siemens)	3	Gadopentetate dimeglumine	0.15	Look–locker	N/A
Tham et al 2013	Sonata (Siemens)	1.5	Gadopentetate dimeglumine	0.125	SASHA	15

*MRI*, magnetic resonance imaging; *MOLLI*, modified look-locker inversion recovery; *SASHA*, saturation recovery single-shot acquisition

### Extracellular volume

**Fig. 2:**
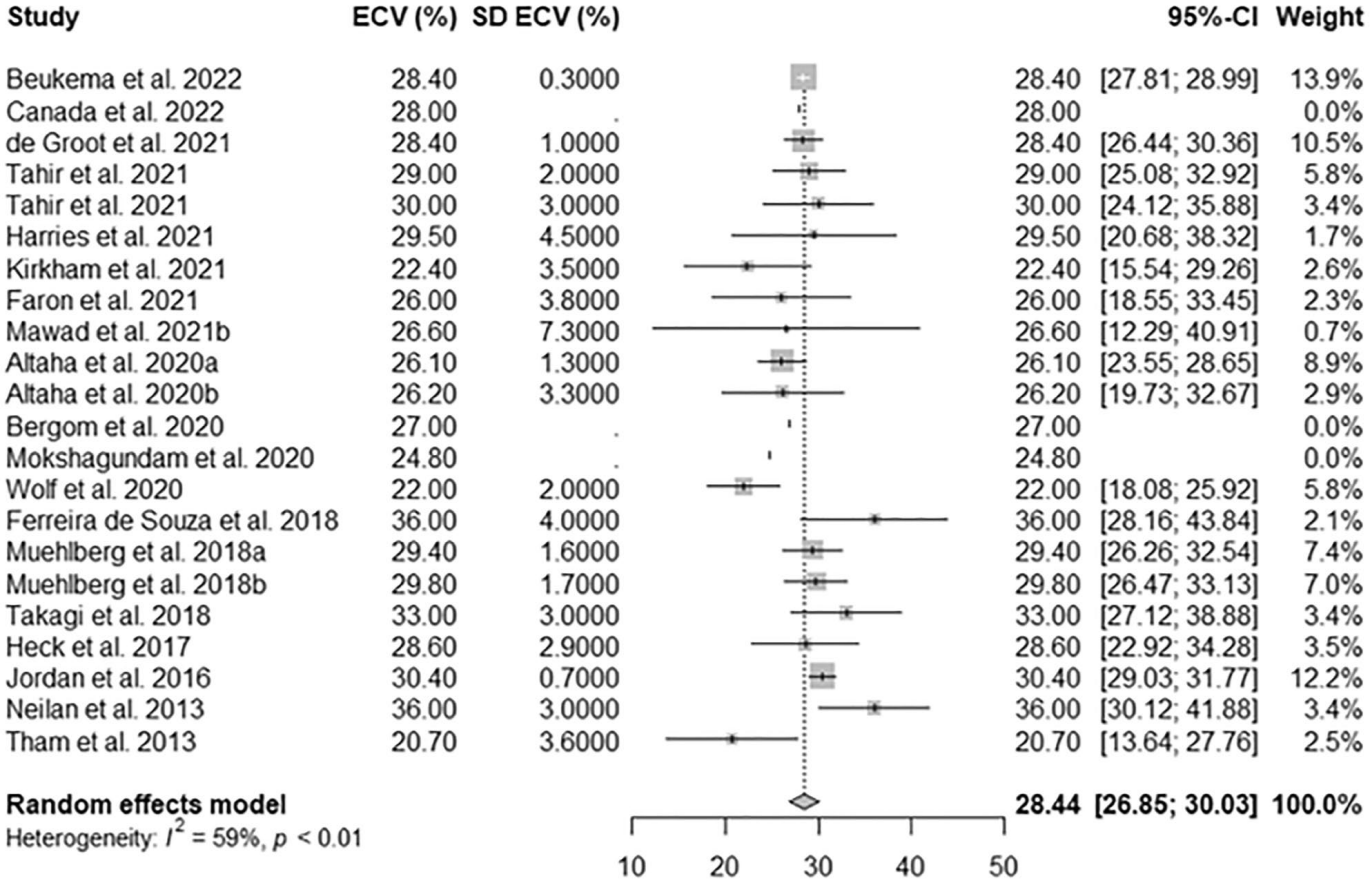
Forest plot for pooled myocardial extracellular volume (ECV) in subjects who underwent cardiotoxic cancer therapy among included works. SD, standard deviation; 95%-CI, 95% confidence interval

**Fig. 3:**
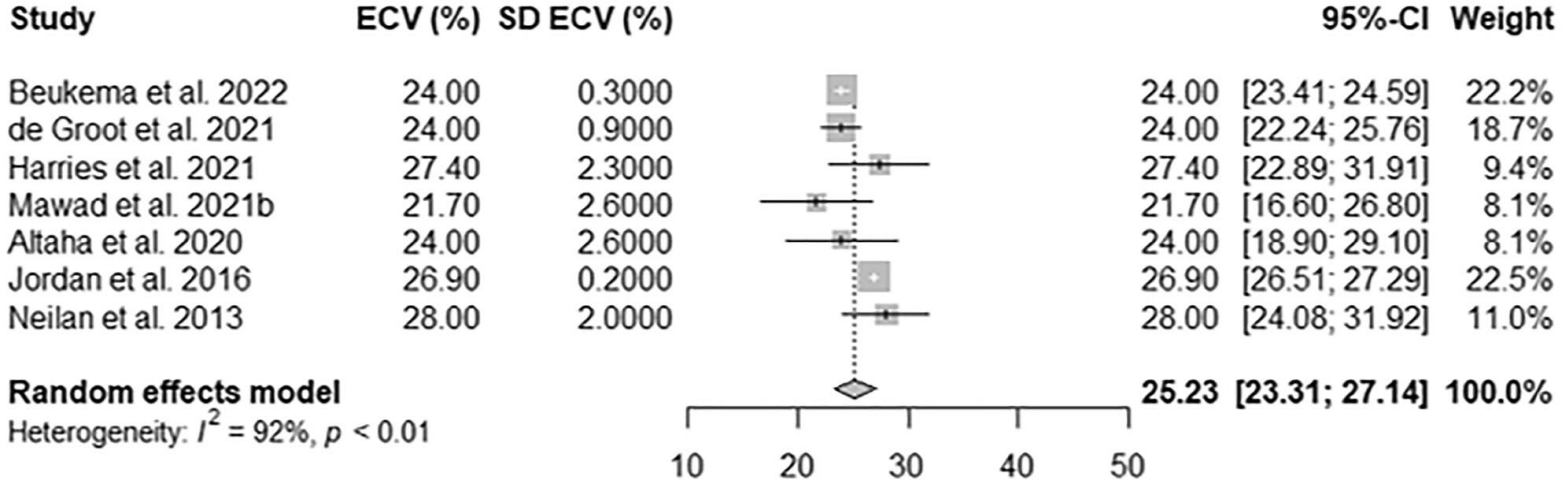
Forest plot for pooled myocardial extracellular volume (ECV) in controls who did not undergo any cardiotoxic cancer therapy among included works. SD, standard deviation; 95%-CI, 95% confidence interval

Pooled ECV was 28.44% (95% confidence interval, CI, 26.85 − 30.03%) among subjects who had undergone cardiotoxic cancer therapy, whereas it was 25.23% (95%CI 23.31 − 27.14%) among those who had not, the former being significantly higher (*p* = .003) than the latter. Forest plots for both groups are shown in Figs. 2 and 3.

Overall, only 7 studies included both cases and matched controls [22, 24, 26, 31, 33, 34, 38], leading to a pooled standardized difference of 1.16% (95%CI 0.64−1.69%).

Among clinical and technical variables, only magnetic field strength (*p* = .006) and the sequence used for T1 mapping (*p* = .02) yielded a significant impact on ECV values, whereas sex (*p* = .87), patients’ age (*p* = .19), type of cancer (*p* = .10), MRI unit (*p* = .08), contrast agent type (*p* = .64) or dose (*p* = .21), and contrast timing (*p* = .77) did not. In addition, there was no significant correlation between ECV and MRI-derived LVEF (*p* = .32). There were no differences in magnetic field strength (*p* = .64), or sequence used for T1 mapping (*p* = .99) between those who underwent cardiotoxic cancer therapy and those who did not.

Among patients who underwent cardiotoxic treatments, pooled ECV was similar (*p* = .70) in subjects who displayed overt cardiac dysfunction (29.05%, 95%CI 10.42−47.67%), and those who did not (28.40%, 95%CI 26.61−30.19%).

Concerning different cardiotoxic treatment regimens, pooled ECV was 28.50% (95%CI 26.44−30.56%) for chest radiotherapy combined with non-cardiotoxic chemotherapy, 29.00% (95%CI 25.08−32.92%) for chest radiotherapy combined with anthracyclines, 30.00% (95%CI 24.12−35.88%) for chest radiotherapy alone, 28.92% (95%CI 25.55−32.30%) for anthracyclines alone, 25.72% (95%CI 22.23−29.20%) for anthracyclines combined with antibodies, and 26.00% (95%CI 18.55−33.45%) for antibodies alone, with the difference among treatment schemes leaning towards statistical significance (*p* = .06).

### Quality assessment

Methodological quality of the studies according to the QualSyst tool showed low risk of bias and is summarized in Supplementary Material 2.

### Publication bias

**Fig. 4:**
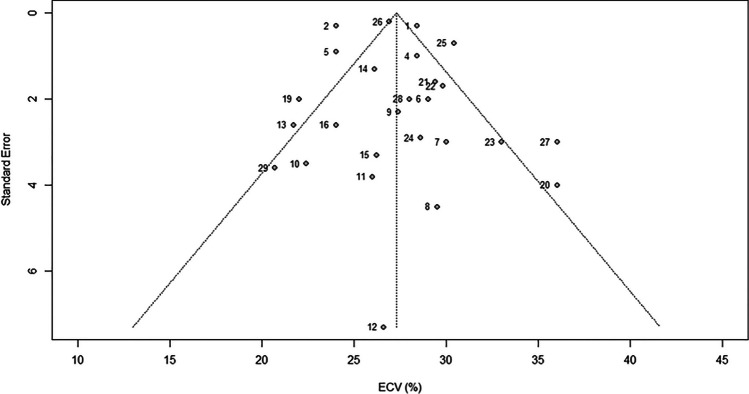
Funnel plot outlining the risk of publication bias

The Egger test did not indicate any risk of publication bias among included studies (*p* = .54), and neither did the funnel plot displayed in Fig. 4.

## Discussion

We observed an increase in ECV consistent across all meta-analyzed studies assessing patients who underwent cardiotoxic cancer therapy, most studies relating increases directly to treatment doses [23, 29, 38, 39]. In fact, pooled ECV among patients subject to cardiotoxic treatment regimens was found to be significantly higher (28.44%, 95%CI 26.85−30.03%) than pooled ECV among those who had not (25.23%, 95%CI 23.31−27.14%, *p* = .003), on the higher end of normal reference values [41]. Similarly, the standardized mean difference observed in studies presenting a case-control design was not negligible (1.16%, 95%CI 0.64−1.69%), and such a difference was expected, as the primary mechanism of dose-related cardiotoxicity, such as that of anthracyclines and chest radiotherapy, is cardiomyocyte death via necrosis or apoptosis, leading to myocardial fibrosis [42].

Moreover, post-chemotherapy ECV values were elevated both in patients with normal LVEF [26, 37, 39] and in those with decreased LVEF [22, 30, 31], with no statistically significant correlation between ECV and MRI-derived LVEF (*p* = .32). This important finding supports a potential application of ECV for the detection of not only overt, but also subtle and early changes in myocardial composition, which may not be functionally evident through LVEF monitoring, due to cardiac compensation mechanisms.

Regarding technical variables, magnetic field strength (*p* = .006) and the sequence used for T1 mapping (*p* = .02) yielded a significant impact on ECV values, with most studies using modified look-locker inversion recovery (MOLLI) sequences on 1.5-T MRI units from varying manufacturers. As ECV is calculated by considering the change in T1 relaxivity before and after contrast administration, rather than T1 absolute values, it is more reproducible, as long as consecutive measurements are performed on the same MRI unit [43]. Regarding different treatment regimens, pooled ECV values did not vary significantly according to treatment scheme albeit leaning towards significance (*p* = .06), supporting the fact that both chest radiotherapy and anthracyclines ultimately lead to myocardial fibrosis, while the stochastic cardiotoxicity of antibodies may yield a lesser impact on ECV values [23, 38].

In prior literature, ECV has also shown correlations with patient prognosis [44] and may therefore provide additional clinical information. Moreover, in addition to MRI, recent works proposed that the evaluation of ECV could also be performed on CT scans [45]. This approach may prove advantageous, as chest CT is already included in the diagnostic algorithm and in the follow-up of many different neoplasms [46]. CT-derived ECV has shown strong correlations to MRI-derived ECV [47]; thus, findings related to the role of ECV in monitoring cancer therapy–related cardiotoxicity may potentially translate from MRI to CT, and the two modalities could also be used interchangeably according to clinical needs. For instance, previous studies have shown that myocardial ECV, assessed at non-gated contrast-enhanced CT, rises significantly in breast cancer patients undergoing anthracycline-based regimens [48] and in patients with esophagus cancer treated with chest radiotherapy [49]. In this sense, while it might not be realistic to screen each patient undergoing cancer treatment for cardiotoxicity using MRI, MRI could be reserved to high-risk patients, such as those with previous comorbidities, undergoing therapies such as anthracyclines or radiation therapy, which are known to yield a dose-dependent effect [50]. Conversely, once the potential role of ECV as an early biomarker of cardiotoxicity is established, patients who already undergo CT as a part of their clinical pathway, regardless of their treatment regimen, could be screened for cardiotoxicity via CT-derived ECV.

Our study presents some limitations. First, the works included in our meta-analysis displayed some degree of heterogeneity concerning clinical characteristics and technical aspects of ECV analysis. In fact, despite anthracyclines representing most of the treatment regimens studied in association to cardiotoxicity, the study groups included in the review underwent cancer therapy for different neoplasms and thus received slightly different regimens. Moreover, even though most studies were carried out using MOLLI sequences on 1.5-T units, ECV was assessed with different MRI units and different contrast agents. Follow-up timings were also heterogeneous; nevertheless, we only included follow-up timings longer than 3 months from cardiotoxic treatment, to ensure that rises in ECV were due to fibrosis instead of residual inflammation. Additionally, not all the studies performed a longitudinal assessment of ECV, lacking data regarding clinical outcomes and pre-treatment ECV values. Furthermore, data reporting treatment doses and regimens was somewhat heterogeneous, and did not allow the performance of meta-regression analyses to review whether cardiotoxicity was dose-dependent. Nevertheless, we know from previous literature that anthracyclines, along with radiotherapy, present with type 1 cardiotoxicity according to Ewer, which is dose-dependent and irreversible, whereas antibodies present with type 2, which is stochastic and may be reversible to a certain extent [50]. Last, while our analysis did include a mixture of retrospective and prospective studies, only two included works actually presented a retrospective design, accounting for 124/1123 patients (11%). As such, even considering the inherent source of bias delivered by retrospective study designs, we do not expect such issue to yield a considerable impact on the results from our meta-analysis.

Future prospective studies may be conducted to determine to what extent ECV monitoring may help prevent, identify, and treat cancer therapy–induced cardiotoxicity. Cardiac MRI might be performed before starting cancer therapy to obtain baseline reference values for each patient, and then at predetermined intervals during and after treatment, and at follow-up. More so, clinical events should be registered, so to potentially find a minimum ECV variation related to clinical adverse outcomes. Expanding on the research of Heck et al [39], integration of ECV monitoring in clinical trials assessing the effects of cardioprotective agents, such as angiotensin-II-receptor antagonists and beta blockers, could shed light on potential ECV thresholds for prevention of cardiotoxicity at a very early stage.

In conclusion, the higher pooled ECV in patients who underwent cardiotoxic treatment could reflect subclinical changes in myocardial structure associated to cancer therapy, suggesting a role for ECV as an early biomarker of cardiotoxicity. Further studies with larger samples, more standardized clinical/technical parameters, and follow-up timings are warranted to identify specific reference values that indicate the occurrence of cardiac changes related to cardiotoxicity, while a patient-centered approach (with cardiac MRI before, during, and after therapy) could support a step forward in personalizing type and regimens of anticancer therapy. An ECV-based detection of high-risk patients could allow the implementation of measures to prevent overt cardiac pathology.

### Supplementary Information

Below is the link to the electronic supplementary material.

Supplementary file1 (PDF 164 KB)

## Abbreviations

CT: Computed tomography
CTRCD: Cancer therapy–related cardiac dysfunction
ECV: Extracellular volume
EMBASE: Excerpta Medica dataBASE
ESC: European Society of Cardiology
LVEF: Left ventricular ejection fraction
MeSH: Medical Subject Headings
MOLLI: Modified look-locker inversion recovery
MRI: Magnetic resonance imaging
PICO: Problem, Intervention, Comparison, Outcome
PRISMA: Preferred Reporting Items for Systematic reviews and Meta-Analyses
RT: Radiation therapy
SASHA: Saturation recovery single-shot acquisition
SD: Standard deviation

## Declarations

### Guarantor

The scientific guarantor of this publication is Francesco Sardanelli.

### Statistics and biometry

One of the authors has significant statistical expertise.

### Study subjects or cohorts overlap

To the best of our knowledge, there is no overlap between subjects reported in the included works, and measures were taken to minimize the chance of this occurrence.

### Methodology

• Meta-analysis

• Multicentre study
