# Supplementary material for: MRI-derived extracellular volume as a biomarker of cancer therapy cardiotoxicity: systematic review and meta-analysis
Source: Eur Radiol. 2023 Oct 12;34(4):2699–710. doi: 10.1007/s00330-023-10260-8 (PMC10957707; doi:10.1007/s00330-023-10260-8)
Supplement: Supplementary file 1 — Supplementary file1 (PDF 164 KB) [file 330_2023_10260_MOESM1_ESM.pdf]

MRI-derived extracellular volume as a biomarker of cancer therapy cardiotoxicity: Systematic review and meta-analysis

Electronic Supplementary Material

Supplementary Material 1: Search string.

Update: November 15th, 2022

Databases used: MEDLINE (PubMed), EMBASE (Elsevier), Web of Science (Clarivate Analytics), and the Cochrane Library databases (Cochrane Database of Systematic Reviews and Cochrane Central Register of Controlled Trials).

A controlled vocabulary (medical subject headings in PubMed and EMBASE thesaurus keywords in EMBASE) was used. The search string was built using the following strategy, based on the PICO model:

'extracellular space'/exp + synonyms

'cardiovascular magnetic resonance'/exp + synonyms

'chemotherapy'/exp OR 'radiotherapy'/exp + synonyms

'cardiotoxicity'/exp + synonyms

Full search strings

The full search string for EMBASE (Elsevier) was:

('extracellular space'/exp OR 'extracellular fluid space' OR 'extracellular fluid volume' OR 'extracellular mass' OR 'extracellular space' OR 'extracellular volume' OR 'extracellular water volume' OR 'space, extracellular' OR ecv OR 'cardiac biomarker'/exp OR 'cardiovascular biomarker' OR 'myocardial extracellular volume'/exp) AND ('cardiovascular magnetic resonance'/exp OR 'cmr (cardiovascular magnetic

resonance)' OR 'cardiac magnetic resonance' OR 'cardiac magnetic resonance imaging' OR 'cardiovascular mri' OR 'cardiovascular magnetic resonance' OR 'cardiovascular magnetic resonance imaging' OR 'nuclear magnetic resonance imaging'/exp OR 'mri' OR 'nmr imaging' OR 'imaging, magnetization transfer' OR 'magnetic resonance imaging' OR 'magnetic resonance tomography' OR 'magnetization transfer imaging' OR 'mr imaging' OR 'nuclear magnetic resonance imaging' OR 'cardiac mri') AND ('chemotherapy'/exp OR 'chemotherapeutics' OR 'chemotherapy' OR 'drug therapy'/exp OR 'drug therapy' OR 'drug treatment' OR 'medicament therapy' OR 'medicament treatment' OR 'medication' OR 'medicinal therapy' OR 'medicinal treatment' OR 'pharmaceutical therapy' OR 'pharmaceutical treatment' OR 'pharmaco-therapy' OR 'pharmaco-treatment' OR 'pharmacological therapy' OR 'pharmacological treatment' OR 'pharmacotherapy' OR 'pharmacotreatment' OR 'therapeutic uses' OR 'therapy, drug' OR 'therapy, pharmacological' OR 'treatment, drug' OR 'treatment, pharmacological' OR 'radiotherapy'/exp OR 'bioradiant therapy' OR 'bucky irradiation' OR 'bucky radiation' OR 'bucky radiotherapy' OR 'bucky ray' OR 'bucky ray radiation' OR 'bucky therapy' OR 'fractionated radiotherapy' OR 'hemibody irradiation' OR 'hypophysectomy, radiation' OR 'hypophysis irradiation' OR 'hypophysis radiation' OR 'irradiation therapy' OR 'irradiation treatment' OR 'irradiation, hypophysis' OR 'lymphatic irradiation' OR 'pituitary irradiation' OR 'radiation beam centration' OR 'radiation repair' OR 'radiation therapy' OR 'radiation treatment' OR 'radio therapy' OR 'radio treatment' OR 'radiohypophysectomy' OR 'radiology, therapeutic' OR 'radiotherapy' OR 'radiotreatment' OR 'roentgen irradiation, therapeutic' OR 'roentgen therapy' OR 'roentgen treatment' OR 'rontgen therapy' OR 'therapeutic radiology' OR 'therapy, irradiation' OR 'therapy, radiation' OR 'therapy, roentgen' OR 'treatment, irradiation' OR 'treatment, radiation' OR 'treatment, roentgen' OR 'x radiotherapy' OR 'x ray therapy' OR 'x ray treatment' OR 'x-ray therapy' OR 'cancer radiotherapy'/exp OR 'cancer irradiation therapy' OR 'cancer radiation' OR 'cancer radiation therapy' OR 'cancer radiation, therapeutic' OR 'cancer radiotherapy' OR 'radiotherapy, cancer' OR 'tumor irradiation' OR 'tumor radiation' OR 'tumor radiotherapy' OR 'tumour irradiation' OR 'tumour radiation' OR 'tumour radiotherapy' OR 'intensity modulated radiation therapy'/exp OR 'imrt' OR 'intensity modulated arc therapy' OR 'intensity

modulated photon radiotherapy' OR 'intensity modulated radiation therapy' OR 'intensity modulated radiotherapy' OR 'intensity modulated therapy' OR 'intensity-modulated radiation therapy' OR 'intensity-modulated radiotherapy' OR 'radiotherapy, intensity modulated' OR 'radiotherapy, intensity-modulated') AND ('cardiotoxicity'/exp OR 'cardio toxicity' OR 'cardiotoxic effect' OR 'cardiotoxicity' OR 'cardiotoxicology' OR 'heart toxicity' OR 'toxicity, heart' OR 'cardiac toxicity')

The full search string for PubMed (MEDLINE) was:

1 TOPIC: (Extracellular volume)

(((((extracellular space[MeSH Terms]) OR (extracellular spaces[MeSH Terms])) OR (ECV[MeSH Terms])) OR (ECV)) OR (Extracellular mass)) OR (myocardial extracellular volume)

2 TOPIC: (Magnetic resonance imaging)

((((((magnetic resonance imaging[MeSH Terms]) OR (Cardiac magnetic resonance imaging[MeSH Terms])) OR (CMR[MeSH Terms])) OR (MRI[MeSH Terms])) OR (cardiovascular MRI[MeSH Terms])) OR (nuclear magnetic resonance imaging[MeSH Terms])) OR (NMRI[MeSH Terms])

3 TOPIC: (Chemotherapy and Radiotherapy)

(((((((Chemotherapy[MeSH Terms]) OR (Radiotherapy[MeSH Terms])) OR (adjuvant chemotherapy[MeSH Terms])) OR (adjuvant radiotherapy[MeSH Terms])) OR (adjuvant radiotherapies[MeSH Terms])) OR (treatment drug[MeSH Terms])) OR (drug treatment center[MeSH Terms])) OR (irradiation[MeSH Terms])

4 TOPIC: (Cardiotoxicity)

(((cardiotoxicity[MeSH Terms]) OR (cardiac toxicity[MeSH Terms])) OR (cardio toxicity[MeSH Terms])) OR (cardiotoxic effect[MeSH Terms])

The full search string for Web of Science (Clarivate Analytics) was:

((((((((((KP=(Extracellular volume)) OR KP=(Extracellular space)) OR KP=(ECV)) OR KP=(myocardial extracellular volume)) AND KP=(magnetic resonance imaging)) OR KP=(Cardiac MRI)) OR KP=(cardiovascular MRI)) OR KP=(Cardiac magnetic resonance imaging)) AND KP=(Chemotherapy)) OR KP=(Radiotherapy)) AND KP=(Cardiotoxicity)

The full search string for Cochrane Library (Cochrane) was:

"Extracellular volume" OR "Extracellular space" in Title Abstract Keyword AND "Magnetic resonance imaging" in Title Abstract Keyword AND "Chemotherapy" OR "Radiotherapy" in Title Abstract Keyword AND "Cardiotoxicity" in Title Abstract Keyword AND "Biomarker" in Title Abstract Keyword - (Word variations have been searched)

Supplementary Material 2: Results from quality assessment according to the QualSyst tool.

Criteria	Beukema et al. 2022	Canada et al. 2022	de Groot et al. 2021	Tahir et al. 2021	Harries et al. 2021	Kirkham et al. 2021	Faron et al. 2021	Mawad et al. 2021	Altaha et al. 2020	Bergom et al. 2020	Mokshagundam et al. 2020	Wolf et al. 2020	Ferreira de Souza et al. 2018	Muehlberg et al. 2018	Takagi et al. 2018	Heck et al. 2017	Jordan et al. 2016	Neilan et al. 2013	Tham et al. 2013
Question/objective sufficiently described?	2	2	2	2	2	2	2	2	2	2	2	2	2	2	2	2	2	1	2
Study design evident and appropriate?	2	2	2	2	2	2	2	2	2	2	2	1	2	2	2	2	2	1	2
Context for the study clear?	2	2	2	2	1	2	2	2	2	2	2	2	2	2	2	2	2	2	2
Connection to a theoretical framework/wider body of knowledge?	1	2	2	1	2	2	2	2	2	2	1	1	2	1	2	2	1	1	2
Sampling strategy described, relevant, and justified?	1	1	2	1	1	2	1	1	2	1	1	1	1	1	1	2	1	1	1
Data collection methods clearly described and systematic?	1	2	2	1	1	2	2	1	2	2	1	1	2	1	2	2	1	1	1
Data analysis clearly described and systematic?	2	2	2	2	1	2	2	1	2	2	1	2	2	1	2	2	1	2	1
Use of verification procedure(s) to establish credibility?	1	1	1	1	1	1	1	1	1	1	1	1	1	1	1	1	1	1	1
Conclusions supported by the results?	1	2	2	1	1	2	2	2	2	2	2	2	2	2	2	2	2	2	1
Reflexivity of the account?	1	1	1	2	2	2	2	2	2	2	1	2	2	1	2	2	1	1	1
**Total**	**14**	**17**	**18**	**15**	**14**	**19**	**18**	**16**	**19**	**18**	**14**	**15**	**18**	**14**	**18**	**19**	**14**	**13**	**14**
**Overall (%)**	**70%**	**85%**	**90%**	**75%**	**70%**	**95%**	**90%**	**80%**	**95%**	**90%**	**70%**	**75%**	**90%**	**70%**	**90%**	**95%**	**70%**	**65%**	**70%**
